# Serum HCoV-spike specific antibodies do not protect against subsequent SARS-CoV-2 infection in children and adolescents

**DOI:** 10.1016/j.isci.2023.108500

**Published:** 2023-11-21

**Authors:** Helen Ratcliffe, Karen S. Tiley, Stephanie Longet, Claire Tonry, Cathal Roarty, Chris Watson, Gayatri Amirthalingam, Iason Vichos, Ella Morey, Naomi L. Douglas, Spyridoula Marinou, Emma Plested, Parvinder K. Aley, Eva Galiza, Saul N. Faust, Stephen Hughes, Clare Murray, Marion R. Roderick, Fiona Shackley, Sam Oddie, Tim W.R. Lee, David P.J. Turner, Mala Raman, Stephen Owens, Paul J. Turner, Helen Cockerill, Jamie Lopez Bernal, Samreen Ijaz, John Poh, Justin Shute, Ezra Linley, Ray Borrow, Katja Hoschler, Kevin E. Brown, Miles W. Carroll, Paul Klenerman, Susanna J. Dunachie, Mary Ramsay, Merryn Voysey, Thomas Waterfield, Matthew D. Snape

**Affiliations:** 1 Centre for Clinical Vaccinology and Tropical Medicine, University of Oxford, Oxford, UK; 2 Wellcome Centre for Human Genetics, Nuffield Department of Medicine, University of Oxford, Oxford, UK; 3 Wellcome-Wolfson Institute for Experimental Medicine, Queen’s University Belfast- School of Medicine, Dentistry and Biomedical Sciences, Belfast, UK; 4 UK Health Security Agency; 5 St Georges Hospital NHS Foundation Trust; 6 NIHR Southampton Clinical Research Facility, University Hospital Southampton NHS Foundation Trust and Faculty of Medicine and Institute of Life Sciences, University of Southampton; 7 National Immunisation Schedule Evaluation Consortium; 8 Manchester University NHS Foundation Trust, NIHR Manchester Biomedical Research Centre, Manchester Academic Health Science Centre, Manchester, UK; 9 Division of Infection, Immunity and Respiratory Medicine, School of Biological Sciences, University of Manchester, Manchester, UK; 10 University Hospitals Bristol NHS Foundation Trust; 11 Sheffield Children’s Hospital NHS Trust; 12 Bradford Teaching Hospitals NHS Foundation Trust; 13 Leeds Teaching Hospitals NHS Trust; 14 School of Life Sciences, University of Nottingham; 15 Nottingham University Hospitals NHS Trust; 16 University Hospitals Plymouth NHS Trust; 17 The Newcastle Upon Tyne Hospitals NHS Foundation Trust; 18 National Heart & Lung Institute, Imperial College London; 19 West Suffolk NHS Foundation Trust; 20 Translational Gastroenterology Unit, University of Oxford, Oxford, UK; 21 National Institute for Health Research (NIHR) Oxford BRC; 22 Centre for Tropical Medicine and Global Health, Nuffield Department of Clinical Medicine, University of Oxford, Oxford, UK

**Keywords:** Health sciences, Medicine, Medical specialty, Immunology, Virology

## Abstract

SARS-CoV-2 infections in children are generally asymptomatic or mild and rarely progress to severe disease and hospitalization. Why this is so remains unclear. Here we explore the potential for protection due to pre-existing cross-reactive seasonal coronavirus antibodies and compare the rate of antibody decline for nucleocapsid and spike protein in serum and oral fluid against SARS-CoV-2 within the pediatric population. No differences in seasonal coronaviruses antibody concentrations were found at baseline between cases and controls, suggesting no protective effect from pre-existing immunity against seasonal coronaviruses. Antibodies against seasonal betacoronaviruses were boosted in response to SARS-CoV-2 infection. In serum, anti-nucleocapsid antibodies fell below the threshold of positivity more quickly than anti-spike protein antibodies. These findings add to our understanding of protection against infection with SARS-CoV-2 within the pediatric population, which is important when considering pediatric SARS-CoV-2 immunization policies.

## Highlights

• Anti-spike antibodies against HCoV do not protect against SARS-CoV-2 infection
• Post SARS-CoV-2 infection, HCoV betacoronaviruses anti-spike antibodies are boosted
• Antibodies against spike persist for longer than nucleocapsid for SARS-CoV-2

## Graphical abstract

**Figure:**
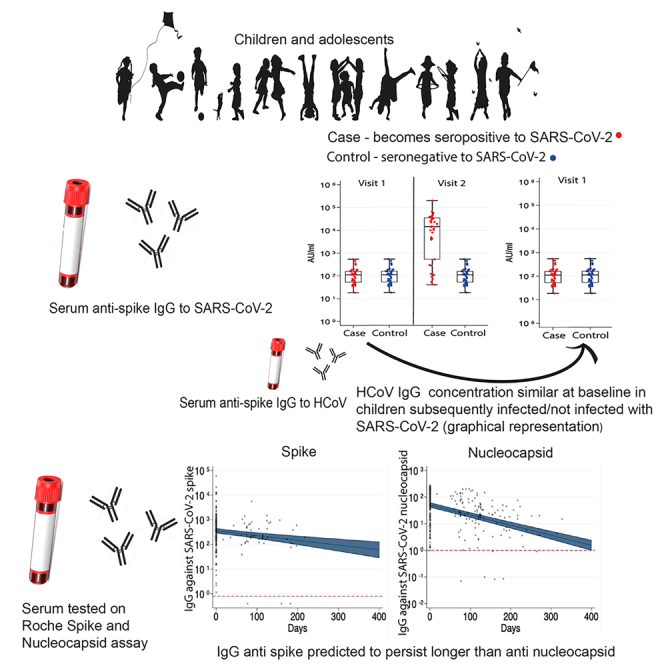

## Introduction

Since the emergence of SARS-CoV-2, it has been noted that infections experienced by children were generally asymptomatic and rarely progressed to severe disease and hospitalisation.^1^ Understanding why children are less severely affected is important to further our knowledge of COVID-19 pathogenesis. Hypotheses to date have included differences in humoral immune responses to SARS-CoV-2 infections in adults and children or differences in baseline seasonal coronavirus humoral immunity.^2^ Alternate theories suggest differences in cellular immunity such as T cell responses or cytokine responses in adults and children.^1^^,^^3^

Uncertainty remains regarding the role of immunity against seasonal coronaviruses in providing protection against SARS-CoV-2 infections.

There are two families of seasonal coronaviruses: alphacoronoviruses (229E and NL63) and betacoronaviruses (OC43, HKU-1). Dowell et al. showed SARS-CoV-2 infection in children boosts humoral responses against alphacoronaviruses (229E and NL63) and betacoronaviruses (OC43, HKU-1)^4^ and that concentrations of antibodies against OC43 and HKU-1 following SARS-CoV-2 infection were significantly higher in children than adults.^4^ The implications of these findings are unclear, as it could not be determined if the observed differences pre-dated the SARS-CoV-2 infection. Furthermore, given antibodies to 229E, NL63, HKU-1, and OC43 viruses provide only short-term immunity from reinfection with the same virus,^5^^,^^6^^,^^7^ any cross-reactive protection may be short-lived.

There is also uncertainty about the persistence of immune responses post SARS-CoV-2 infection in young children and adolescents, as most available data have been derived from (predominantly adult) severe or hospitalized cases or from population serosurveys where most participants are adults or from vaccination studies.

**Figure 1:**
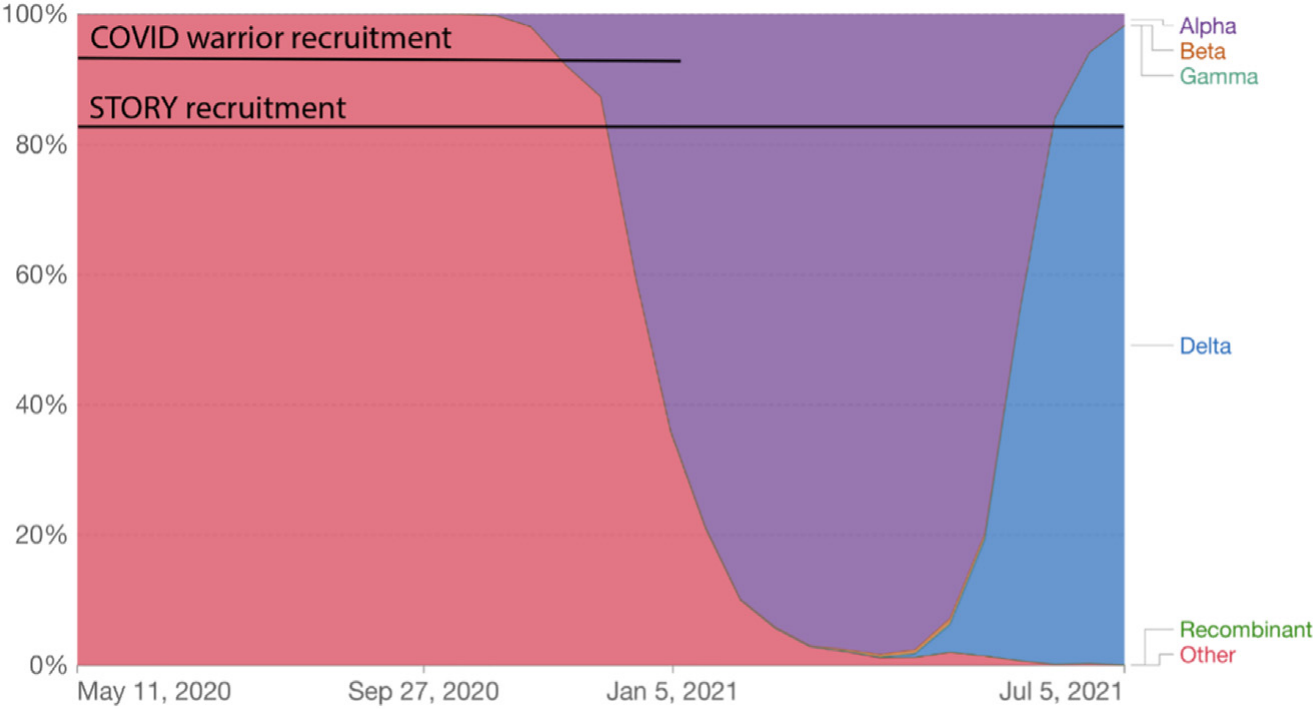
Timeline of SARS-CoV-2 variants circulating within the UK up until July 2021, adapted to show recruitment periods for “STORY” and “COVID Warrior” studies This figure was produced by my world data and licensed under CC BY.

To better understand immunological responses to SARS-CoV-2 and seasonal coronaviruses and the inter-relationship between the two, we analyzed data from two longitudinal cross-sectional seroprevalence studies ”COVID warriors”^8^ (ClinicalTrials.gov Identifier: NCT04347408) and ”STORY”^9^^,^^10^(ClinicalTrials.gov Identifier: NCT04061382). Both studies were carried out within the UK between October 2019 and June 2021 when widespread immunization within the pediatric population was not available (Figure 1)^11^. Strains circulating during this time period included ancestral lineages, alpha, beta and delta variants of SARS-CoV-2.^11^ A case-control analysis was used to determine whether the presence of detectable antibodies against seasonal coronaviruses influenced the subsequent likelihood of seroconversion for SARS-CoV-2. Furthermore, longitudinal data were used to estimate the rate of decline of antibodies against SARS-CoV-2 in serum and oral fluid over time in children.

## Results

### SARS-CoV-2 antibodies in children cross react with beta human coronaviruses (hCov)

**Figure 2:**
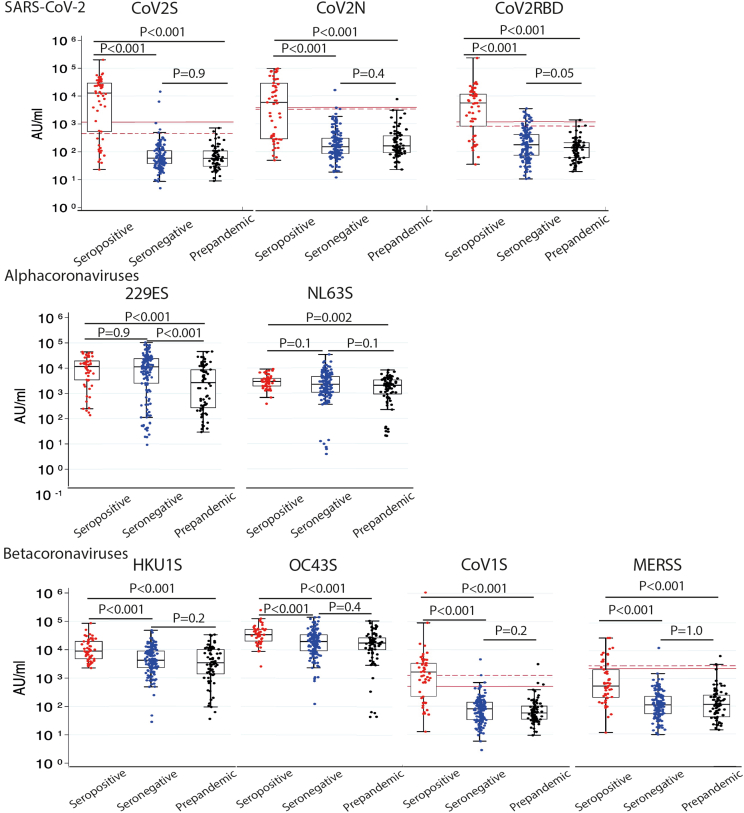
A comparison of seropositive (red), seronegative (blue), and pre-pandemic (black) samples (positivity defined by the original study assays) tested using the MSD platform Mann–Whitney U test was used to compare between groups. A value of p<0.01 were considered statistically significant. The threshold of positivity is shown by a solid line (adult cut-off) and dashed line (pediatric cut-off).

Serum or plasma samples from participants were collected in both studies and were processed using multiple assays evaluating antibodies against spike protein, nucleocapsid and receptor binding domains (Table S1). Samples were classified according to Roche Elecsys Anti-SARS-CoV-2 IgG spike assay (RocheS), Roche Elecsys Anti-SARS-CoV-2 IgG nucleocapsid assay (RocheN), DiaSorin LIAISON SARS CoV-2 S1/S2 IgG assay (DiaSorin) and a UK Health Security Agency in house receptor binding domain assay (UKHSA RBD) into seropositive samples (i.e., a positive result against SARS-CoV-2 as defined by the manufacturers’ definition (Table S1)^12^^,^^13^ on one or more of the assays) and seronegative samples (defined as a negative result against SARS-CoV-2 across all assays). In total, 52 children and adolescents with seropositive samples (range 3–19 years, mean and median 12 years) and 145 children and adolescents with seronegative samples (range 0–19 years, mean 10 years, median 11 years) were analyzed. These were compared with 74 pre-pandemic serum samples collected from children aged 0–13 years (mean 7 years, median 7 years) in 2009/2010 (EudraCT Number: 2009-014719-11) and 2017/2018 (EudraCT Number: 2017-004732-11). All samples were retested using the Meso Scale Discovery (MSD) V-PLEX platform to quantify IgG concentrations to SARS-CoV-2 (receptor binding domain, nucleocapsid and spike protein), alpha coronaviruses (229E and NL63 spike protein) and beta coronaviruses (SARS-CoV-1, MERS, HKU-1, and OC43 spike protein). Groups were compared using the Mann–Whitney U test as data were not normally distributed, and results with a value of p < 0.01 were considered statistically significant due to multiple comparisons. Figure 2 shows one sample per participant categorized as seropositive, seronegative (positivity defined on original study assays) or pre-pandemic. IgG concentrations against SARS-CoV-2 S, N and RBD as determined by the MSD assay were significantly higher in seropositive participants than in seronegative and pre-pandemic cohorts. SARS-CoV-2 seropositive children also had significantly higher antibody titers against all other seasonal coronavirus spike proteins than pre-pandemic cohorts and, for the betacoronaviruses, seropositive children had significantly higher antibody titers than seronegative children (Figure 2).

Figure S1 shows concordance for negative results of 84.4–90.6% across all assays when detecting the same SARS-CoV-2 antigen, with the MSD platform identifying 5.6–9.3% more positive results than the four SARS-CoV-2 assays described above.

### hCoV antibodies do not confer protection against SARS-CoV-2 infection

Pre-existing immune responses to seasonal coronaviruses have been postulated as a reason why children are less severely affected by SARS-CoV-2 infection. Consequently, we conducted a matched case-control analysis of 38 cases and 87 controls aged 2–19 years where samples from two time points were available (baseline and seroconversion visit). A seroconversion ”case” was defined by a positive result (above the thresholds of positivity shown in Table S1) for SARS-CoV-2 on at least one study assay (RocheS, Diasorin, RocheN or RBD) at the second or third visit, following consistently negative previous/prior visit result(s). A control was defined as a child who did not seroconvert for SARS-CoV-2 and was matched on.

1. date of the matched case’s first positive result (date +/− 15 days).
2. date of a negative preceding visit (date +/− 15 days).
3. study site (COVID warriors) or NHS region (STORY).
4. Age (COVID warriors +/− two years) STORY (within an age band +/− two years Figure S2).

Between one and three controls were identified for each case. Each case had a defined baseline (V1) visit followed by a V2 visit (where cases had seroconverted), with the timing of V1 and V2 visits for cases matched as above. Samples were tested on the MSD platform. Conditional logistic regression was used to determine if the seasonal coronavirus antibody’s baseline (V1) level could predict SARS-CoV-2 seroconversion (case/control status), adjusting for study (STORY or COVID warriors).

**Figure 3:**
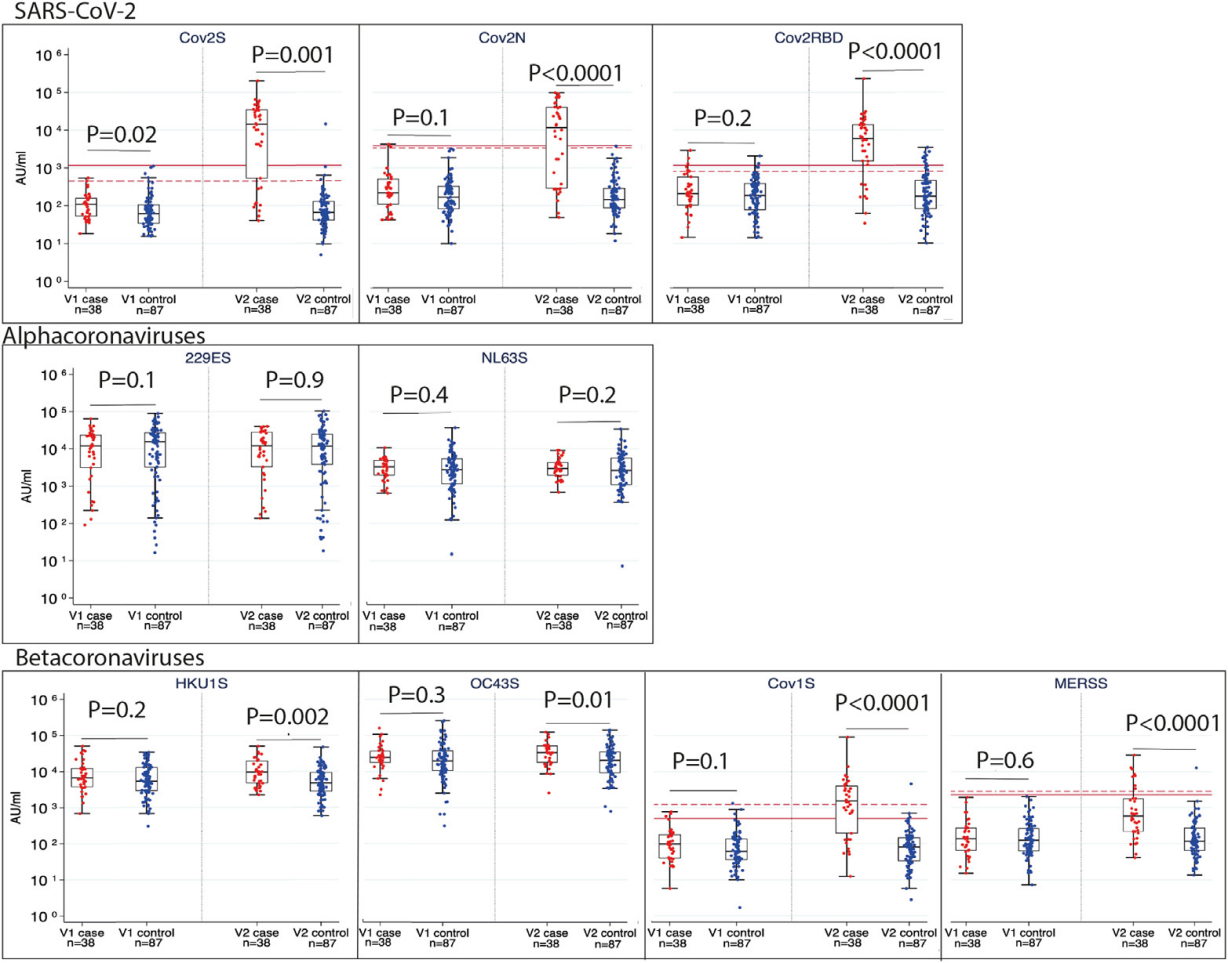
A matched case-control analysis (positivity defined by original study assays) tested on the MSD platform Mann–Whitney U test was used to compare between groups. A value of p < 0.01 were considered statistically significant. The threshold of positivity is shown by a solid line (adult cut-off) and dashed line (pediatric cut-off).

At baseline (V1) cases and controls showed no significant difference in antibody concentrations against any seasonal coronaviruses. Baseline antibodies against seasonal coronaviruses did not alter the risk of being a “case” versus control (Figure 3; Table S2).

At the seroconversion (V2) visit, concentrations of spike-specific IgG were significantly higher in cases than in controls for all SARS-CoV-2 and betacoronavirus HKU1S, Cov1S, MERSS assays (OC43S p = 0.01). No significant differences in alphacoronavirus IgG concentrations between cases and controls were seen at V2 (Figure 3).

### Children are predicted to maintain an SARS-CoV-2 stable immune response to spike protein

Two or more timepoints were available for 1,350 participants across COVID warriors and “STORY” studies. The most frequently used assay was RocheN; 1,263 participants had results at two or more time-points for this assay (Table S3). Seropositive participants from COVID warriors (participants with a positive test on either DiaSorin or RocheN assays) were retested with RocheS to allow the “STORY” and “COVID warrior” datasets to be combined when modeling antibody decline of spike (RocheS) and nucleocapsid (RocheN) antibodies.

Concentrations of SARS-CoV-2 IgG from RocheS and RocheN assays were plotted against time with up to four observations per participant. Time from the first positive antibody result and time from highest positive antibody concentration were explored separately. Participants were grouped by age (0–4 years, 5–9 years, 10–14 years, 15–24 years).

### Anti-nucleocapsid IgG concentrations are predicted to wane more quickly than anti-spike IgG concentrations in serum within the children and adolescent population

**Figure 4:**
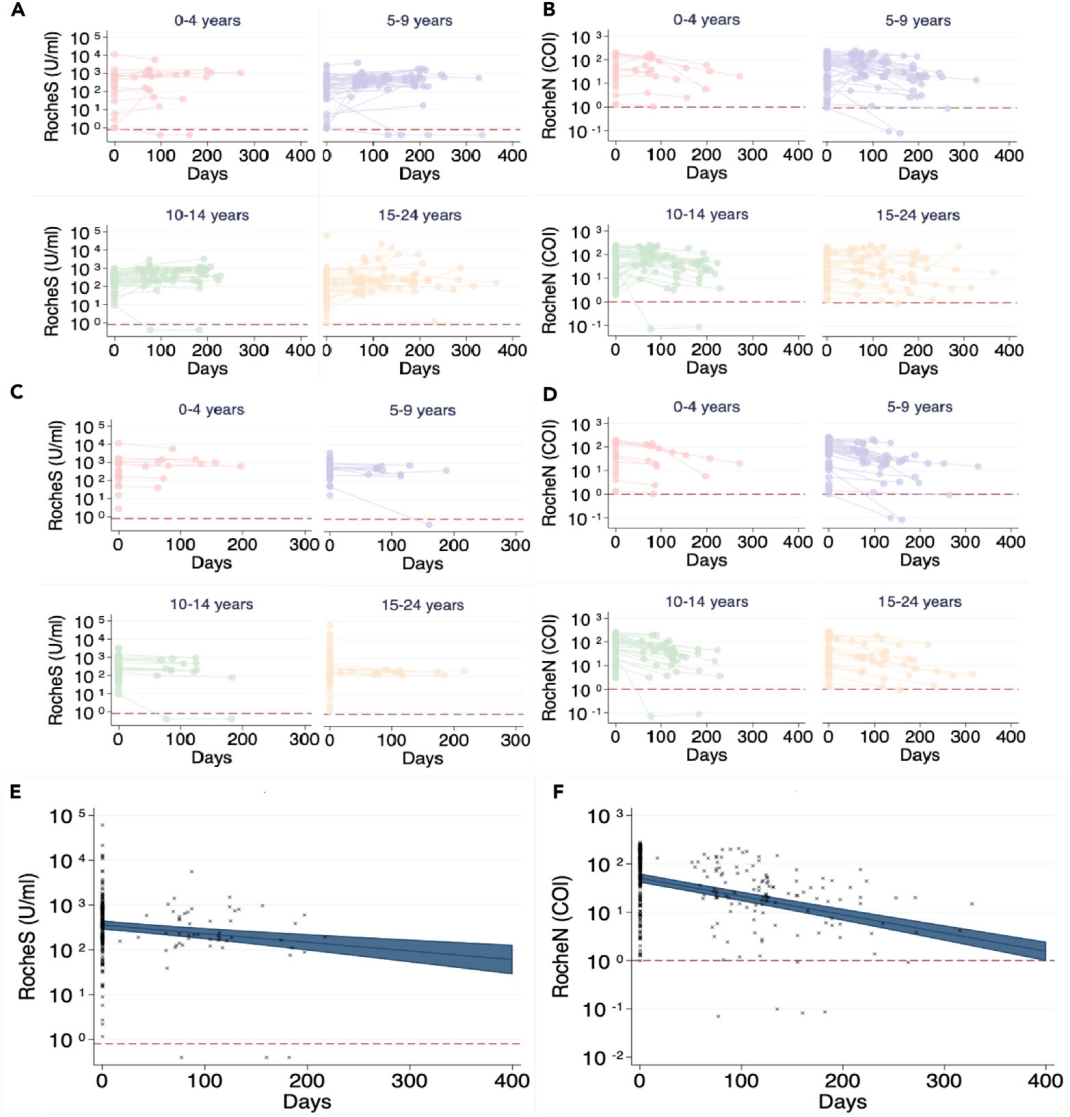
IgG serum antibody concentrations over time Figure shows results of serological testing for (A) Roche Elecsys® Anti-SARS-CoV-2 IgG spike antibody persistence over time. (B) Roche Elecsys® Anti-SARS-CoV-2 IgG nucleocapsid antibody persistence over time. Time-point 0 is defined as the participant’s first positive result (A and B). (C) Roche Elecsys® Anti-SARS-CoV-2 IgG spike antibody persistence over time d. Roche Elecsys® Anti-SARS-CoV-2 IgG nucleocapsid antibody persistence over time. Time-point 0 is defined as the participant’s highest positive result (C and D). Figures (A–D) are broken down into age groups. Figures (E and F) show a mixed regression model predicting the adjusted means with time point 0 being the highest positive result with individual participant's serum antibody concentrations over time marked with (x) for Roche Elecsys® Anti-SARS-CoV-2 IgG spike antibodies (A) and Roche Elecsys® Anti-SARS-CoV-2 IgG nucleocapsid antibodies (B). 95% confidence intervals are shown. The threshold of positivity is indicated by the red line on each plot.

Figures 3A and 3B show antibody titers over time using the RocheS and RocheN assays respectively, where timepoint 0 was defined as the participant’s first positive result using manufacturers’ definitions (Table S1). The spike assay and nucleocapsid assay show a similar trend over all age groups, with IgG subsequently falling below the threshold of detection within the study period for eight out of 175 (4.5%) participants with a positive spike (Figure 4A) compared with six out of 181 (3.3%) participants when measured on the nucleocapsid assay (Figure 4B).

Plotting from the highest positive result for RocheS anti-spike IgG assay (Figure 4C) shows results from 181 participants with one or more samples taken at times ranging between 0 and 217 days (median 0 days, mean, 27 days). The RocheN anti-nucleocapsid IgG assay (Figure 4D) model shows 175 participants with one or more samples taken at times ranging between 0 and 327 days (median 75 days, mean 80 days) after their highest antibody result.

A mixed-effects linear regression was carried out with log-transformed antibody concentration as the outcome, fixed effects for time from the highest positive result as a continuous variable, age group and study, and random intercepts for each participant.^14^ Figures 4E and 3F demonstrate antibody decline graphically by plotting adjusted means for antibody concentration over time with day 0 as the highest positive result for RocheS and RocheN respectively. The half-life was 160 days (95% CI 79–241 days) for anti-spike antibodies and 82 days (95% CI 57–107 days) for anti-nucleocapsid antibodies. Serum anti-nucleocapsid antibodies sero-revert more rapidly than anti-spike antibodies (Figures 4E and 4F; Table 1).

**Table 1:** A comparison of the predicted antibody decline of anti-spike and anti-nucleocapsid IgG concentrations in serum and oral fluid

		Spike		Nucleocapsid	
		Days (95% CI)	IgG concentration (AU/mL)	Days (95% CI)	IgG concentration (COI)
Serum	Time point 0	0	354.8	0	50.7
	Half-life	160 (79–241)	176.2	82 (57–107)	25.1
	Time to sero-revert from positive to negative	1320 (786–1872)	0.8	450 (392–508)	1.0
		Days	IgG concentration (COI)	Days	IgG concentration (COI)
Oral fluid	Time point 0	0	4.1	0	2.9
	Half-life	161 (84–240)	2.0	162 (109–213)	1.5
	Time to sero-revert from positive to negative	323 (178–463)	1.0	250 (184–317)	1.0

### Oral fluid concentrations of IgG against nucleocapsid wane more quickly than against spike protein and both wane more quickly than serum antibodies within the pediatric population

Oral fluid (OF) samples were collected using Oracol^15^ swabs from participants returning for repeat visits within the “STORY” study. OF contains saliva and gingiva-crevicular fluids which has transudate from serum. This results in IgG and IgM concentrations at approximately 1/800 and 1/400, respectively, of that found in serum.^16^ These were analyzed using an SARS-CoV-2 IgG Enzyme immunoassays (EIA) developed by UKHSA, using SARS-CoV-2 viral nucleoprotein (NP) and spike (S) proteins in IgG isotype capture format.^16^

Serum IgG and oral fluid IgG concentrations collected from the same participants at the same time point were concordant in 623/658 (94.6%) and 610/662 (92.1%) for anti-spike IgG and anti-nucleocapsid IgG respectively. The number of serum-positive results with a negative oral fluid result was 34/658 (5.2%) and 50/662 (7.6%) for anti-spike and anti-nucleocapsid IgG respectively (Figure S3), while only 1 (0.2%) and 2 (0.3%) oral fluid results were positive with negative serum results for anti-spike and anti-nucleocapsid IgG (respectively).

**Figure 5:**
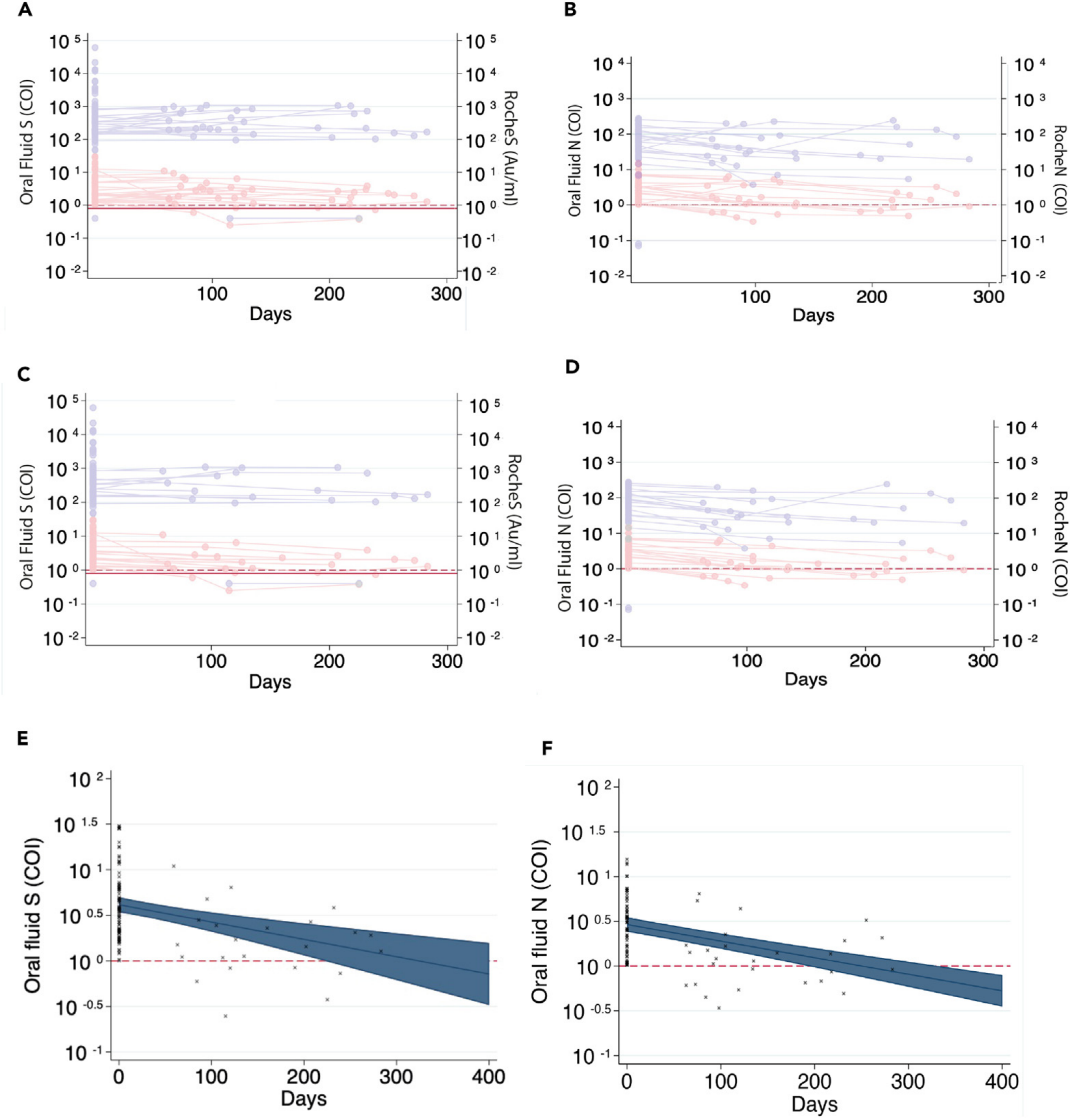
A comparison of IgG antibody concentration in oral fluid and serum samples over time (A–D) Shows both oral fluid (red) and corresponding serum samples where available (blue). (A and B) Where timepoint 0 is defined as the first positive oral fluid result for spike protein (A) and nucleocapsid (B). (C and D) Define time-point 0 as the highest positive oral fluid result for spike protein (C) and nucleocapsid (D). The threshold of positivity is marked by the dashed line (OF) and solid line (serum). These thresholds overlap on nucleocapsid plots. (E and F) Shows a mixed regression model for oral fluid predicting the adjustment means with time point 0 being highest positive result with individual participants antibody concentrations over time marked with (x) for anti-spike protein (E) and anti-nucleocapsid antibodies (F). 95% confidence intervals shown. The threshold of positivity indicated by the red line on each plot.

Figures 5A and 5B shows the change in concentrations over time for oral fluid anti-spike IgG and anti-nucleocapsid IgG from the first positive result. When tested on the oral anti-spike IgG assay 4/26 (15%) of participants who provided at least one result after their initial positive result had concentrations falling below the threshold of detection within the study period compared with 8/20 (40%) falling below the threshold using with oral anti-nucleocapsid IgG assay (Figure 5B).

A mixed-effects linear regression was carried out with antibody concentration as the outcome, fixed effects for time from highest positive result as a continuous variable and age group, and random intercepts for each participant using the same methodology used to assess antibody decline in serum. Antibody decline was demonstrated graphically by plotting adjusted means for antibody concentration at fixed values of days with day 0 defined as the highest positive result.

Timepoint 0 was defined as the highest positive result when calculating adjusted means for antibody titer. The model of oral fluid anti-spike IgG assay contained data from 85 participants with one or more data points taken at times ranging between 0 and 283 days (median 0 days mean 25 days). The oral fluid anti-nucleocapsid IgG assay model contained data from 64 participants with one or more data points taken at times ranging between 0 and 283 days (median 0 days, mean 44 days) after their highest antibody result (Figures 5C and 5D). The models demonstrate that anti-nucleocapsid antibodies in oral fluid are predicted to cross below the threshold of positivity more quickly than anti-spike antibodies but confidence intervals are overlapping (Figures 5E and 5F; Table 1). Oral fluid anti-spike and nucleocapsid, and serum anti-spike antibodies all have a half-life of ∼160 days.

## Discussion

These studies, conducted in a vaccine naive population between the beginning of the pandemic and June 2021 show no evidence of protection against SARS-CoV-2 infection from antibodies against seasonal coronaviruses. Additionally, infected children and teenagers had minimal waning from their highest anti-spike serum IgG antibody concentration (half-life of five months) in the absence of vaccine boosting.

Our data supports and builds on literature which reports a trend of increased antibodies against both alphacoronaviruses and betacoronaviruses in SARS-CoV-2 seropositive versus seronegative participants which was statistically significant for OC43 and HKU-1.^4^ This increase was reported to be due to antibodies that were cross-reactive with the S2 subunit of the spike protein of SARS-CoV-2 which is more closely related in betacoronaviruses.^4^ This concurs with our data demonstrating a significantly higher IgG concentration for betacoronaviruses both when comparing seropositive with seronegative samples, and when analyzing the V2 samples for cases versus controls, but this was not seen with the alphacoronaviruses.

The case-control analysis builds on these cross-sectional comparisons and shows there was no evidence of higher baseline seasonal coronavirus antibodies conveying protection against SARS-CoV-2 infection. This suggests that the higher antibodies against seasonal coronavirus antibodies seen in similar pediatric studies^4^ have occurred in response to SARS-CoV-2 infection, implying cross-reactivity but not cross-protection. A limitation of the case-control analysis is the categorization of participants on different assays before testing on the MSD platform.

The increase in IgG concentration in betacoronaviruses seen within our case-control analysis may be explained by immune imprinting, i.e., the immune system recalling existing memory cells, rather than stimulating *de novo* responses when encountering a novel but closely related antigen (back boosting effect).^17^^,^^18^ In adult patients with severe COVID-19 back boosting of antibodies against the S2 subunit from betacoronaviruses has been recognised,^17^ with Westerhuis et al. specifically demonstrating increases in OC43 specific anti-nucleocapsid and spike antibodies following severe COVID-19 in adults.^19^ The limitation of these studies is that they were in hospitalized patients, but the data we present here suggests this is seen in children with mild infections as well as in adults with severe COVID-19. Lavell et al. studied healthcare workers who were working during the first wave of the pandemic and demonstrated no significant association with HCoV-OC43 spike protein serum IgG concentration and SARS-CoV-2 seropositivity. In contrast, when measuring anti HCoV-OC43 nucleocapsid antibodies, they found that high levels of anti-HCoV-OC43 IgG was an indicator of recent SARS-CoV-2 infection and was associated with protection against SARS-CoV-2 infection.^20^ Our research focused on antibodies against spike protein, and did not evaluate anti-nucleocapsid antibodies or cell-mediated immunity, and these limitations should be considered when drawing conclusions about the association of seasonal coronaviruses with protection against SARS-CoV-2 infection.

While our findings suggest that immunity against seasonal coronaviruses in children does not provide protection against SARS-CoV-2 infections, it is still possible that it could mitigate the severity of infection. There is some evidence for this in adults, in whom higher levels of anti-nucleocapsid IgA antibodies against OC43 were observed in asymptomatic compared with symptomatic SARS-CoV-2 infected participants.^21^ In addition, a higher fold increase in anti-nucleocapsid IgG concentrations against NL63 were seen after asymptomatic versus symptomatic SARS-CoV-2 infections.^21^ It is difficult to distinguish whether the difference in findings between Ortega et al. and our study when looking at baseline anti-HCoV antibodies are due to differences in methodologies or the age groups of the participants. Other adult studies have reported no cross-protection from seasonal coronaviruses against SARS-CoV-2.^22^^,^^23^^,^^24^

The literature shows seropositive children and adults had broadly similar antibody responses against SARS-CoV-2 nucleocapsid. They also demonstrated a trend toward a higher antigen specific geometric mean IgG concentration in children when compared with adults, most notably against spike protein and RBD domains.^4^

The evidence of a lack of cross-protection from seasonal coronaviruses emphasizes the need for ongoing research to understand why children are less susceptible to severe infections, including more detailed assessments of the differences in cellular immune response between adult and pediatric populations. Children are reported to display fewer symptoms than their adult counterparts despite a similar viral load, but they mount a robust innate immune response which allows better early control of inflammation than adult counterparts.^25^

Further clues may be provided by the increase in susceptibility to SARS-CoV-2 following the emergence of omicron variants.^26^ Omicron variants have altered the clinical presentation of COVID-19, predominantly affecting the upper respiratory tract rather than the lower respiratory tract, leading to an increased number of children presenting with croup. Children are more susceptible to upper airway infections as they have relatively smaller airways compared with adults.^27^ The entry of SARS-CoV2 into host cells has also changed with the new variants, with previous strains entering via ACE2 receptors (less numerous in children than adults^28^), while the method of entry for omicron variants is endocytic.^29^ With increased susceptibility to Omicron a proportional increase of severe disease in children has not been seen^26^ suggesting that the method of viral entry was not responsible for the reduced disease severity in children.

Nucleocapsid antibodies waning more quickly than spike antibodies has been widely reported in adults^4^^,^^21^^,^^30^ and in this study, we demonstrate that serum anti-nucleocapsid antibodies serorevert in a shorter time compared with anti-spike antibodies, however, there is no statistically significant difference between half-lives. A limitation of the analysis was there was no way to adjust for reinfections of individuals within the cohort. Therefore, an estimation was made by taking the highest antibody result. In an age of widespread vaccination, serological surveillance of natural infection rates relies on nucleocapsid testing, but our data demonstrate this may underestimate the number of infections within the population in children as well as adults.

An alternative approach to population surveillance is the use of oral fluid, rather than serum, for large-scale population studies in pediatrics as it is more acceptable to parents and young children than a blood test. The usefulness of oral fluid in prevalence calculations is limited as antibody concentrations in gingiva-crevicular fluids, which contains IgG antibodies from serum^31^ have been shown to wane quickly^32^^,^^33^ and therefore would underestimate prevalence. In this study, we demonstrate saliva collection is feasible in younger age groups. Although PCR swabs from the nose and throat were the gold standard during the pandemic, this type of swab was uncomfortable which may impact of the quality of these swabs in younger age groups.^34^ Saliva PCR testing may prove to be more acceptable to young children and therefore be of use in monitoring incidence.^35^^,^^36^^,^^37^^,^^38^^,^^39^

Multiple studies in adults have shown the persistence of antibodies to SARS-CoV-2 infection for up to 18 months regardless of infection severity^30^^,^^40^^,^^41^ however it was noted that participants who were recovering from COVID-19 had significantly reduced neutralizing capacities one year after symptomatic infection.^42^ Dowell et al. assessed the longevity of immune responses for 35 children at 6 months. All had retained humoral immunity and maintained higher antibody concentrations against spike protein and RBD.^4^ By 12 months it was shown that 16 children had antibody responses to spike protein and RBD which were similar but slightly reduced to those levels seen at 6 months.^4^ Studies looking at antibody concentrations over time are further complicated by the emergence of different strains of SARS-CoV-2 and the potential for reinfections within the period of study. “COVID warriors” and “STORY” recruited over periods where ancestral lineages A and B, alpha, beta, gamma and delta were circulating within the UK.^43^ This highlights that surveillance of antibody concentrations in the population does not inform on population immunity.

While we have demonstrated minimal waning in serum anti-spike antibodies against SARS-CoV-2 in children and adolescents, we know this is not protective against breakthrough infections.^44^ As of January 2023, the UKHSA had reported 1.4 million reinfections in England,^44^ with breakthrough infections due to a combination of waning immunity and the emergence of new variants of concern. Therefore, understanding antibody responses in terms of magnitude and decline following natural infection is important as this contributes to population immunity and can therefore inform public health policy and vaccine booster campaigns.

With vaccination programs offering vaccines to all children over five years of age in the UK and with SARS-CoV-2 continuously circulating within the community, the opportunity to study children’s immune responses to SARS-CoV-2 in a naive population becomes more challenging. The data we have presented here are historically unique, providing invaluable insight into the interrelationship between SARS-CoV-2 and seasonal coronaviruses and kinetics of the antibody responses in a predominantly vaccine naive population.

### Limitations of the study

A limitation of the case-control analysis is the lack of a consistent “gold standard” assay to determine SARS-CoV-2 seropositivity. “COVID warriors” and “STORY” used various assays when undertaking seroepidemiological surveys, and participants were categorized as either a case (positive on at least one assay) or a control (negative on all assays). Once these categories had been established, samples were retested using the Meso Scale Discovery assays to compare concentrations of IgG anti-spike protein antibodies against a panel of hCov viruses which included the Meso Scale Discovery SARS-CoV assays. Participants were not recategorized despite discordant Meso Scale Discovery assay results, to maintain adequate sample sizes for analysis. Also, while our analysis suggested that HCoV-spike-specific antibodies do not protect against subsequent SARS-CoV-2 infection however we did not evaluate anti-nucleocapsid antibodies, nor cell-mediated responses and further research should be undertaken to understand the association between the broader immune response to seasonal coronaviruses and SARS-CoV-2 infection.

A limitation of the analysis of antibody response over time is that reinfections were not adjusted for within the cohort, a limitation that was addressed by calculating decline in antibodies from the highest antibody result.

## STAR★Methods

### Key resources table

**Table:** 

REAGENT or RESOURCE	SOURCE	IDENTIFIER
**Biological samples**		

Oxford Vaccine Group Biobank	Oxford Vaccine Group	

**Critical commercial assays**		

Roche Elecsys® Anti-SARS-CoV-2 IgG spike assay	Roche	0928926750
DiaSorin LIAISON® SARS CoV-2 S1/S2 IgG assay	DiaSorin	311510
Roche Elecsys® Anti-SARS-CoV-2 IgG nucleocapsid assay	Roche	09203095501
UKHSA Receptor binding domain (RBD) assay	UKHSA	
MSD SARS-CoV-2 S	Meso Scale Discovery	K15369U-2
MSD SARS-CoV-2 RBD	Meso Scale Discovery	K15369U-2
MSD SARS-CoV-2 N	Meso Scale Discovery	K15369U-2
Oral fluid assays		
SARS-CoV-2 viral nucleoprotein (NP)	UKHSA	
SARS-CoV-2 viral spike (S)	UKHSA	

**Software and algorithms**		

STATA statistical software	Stata	Release 17

### Resource availability

#### Lead contact

Further information and requests for resources and reagents should be directed to and will be fulfilled by the lead contact, Dr. Helen Ratcliffe (helen.ratcliffe@paediatrics.ox.ac.uk).

#### Materials availability

Two pediatric studies supplied samples for analyses. ‘COVID warriors’ obtained ethical approval from the London - Chelsea Research Ethics Committee (REC Reference - 20/HRA/1731) and the Belfast Health & Social Care Trust Research Governance (ref. 19147TW-SW). ‘STORY’ obtained ethical approval from the London-Surrey Research Ethics Committee (REC Reference-19/LO/1040). As part of the ‘STORY’ study, 2963 participants between the ages of 0–24 years provided samples to establish a biobank of serum samples with their vaccination history. This biobank is available through the Oxford Vaccine Group. Samples were collected between October 2019 and June 2021.

#### Data and code availability

• Data reported in this paper will be shared by the lead contact upon request.
• This paper does not report the original code.
• Any additional information required to reanalyse the data reported in this paper is available from the lead contact upon request.

### Experimental model and study participant details

COVID warriors recruited 992 participants from five UK centers between April 2020 and January 2021. Participants were children of healthcare workers (defined as National Health Service (NHS) employee) aged between two and fifteen years at the time of recruitment with 49% of participants identifying as female. Potential participants were recruited via internal intranet advertisements and e-mail circulars.^8^ Participants provided a blood sample at baseline, two and six months.

‘STORY’ was a community-based cross-sectional seroprevalence study recruiting participants aged 0–24 years from 13 sites distributed across all seven NHS regions in England, conducted between October 2019 and June 2021.^9^ The study recruited 2,963 participants 0–24 years, 2,477 of whom were aged 0–18 years. Of the total 2,477, 1,230 individuals (50%) identified as female and 426 (17%) identified as belonging to non-white ethnic groups.^9^

### Method details

Information regarding the study was disseminated by invitation letters sent through Docmail (a UK General Data Protection Regulation compliant bulk mailing system) from extracts provided by either NHS Digital^10^ or Child Health Information Systems (CHIS)^11^ databases, social media campaigns, school nurse newsletters, and pharmacies. Potential participants and their families were invited to visit the study website https://whatsthestory.web.ox.ac.uk for more information and to contact the regional study site if they wished to participate. Once enrolled to the main study participants were asked if they would be happy to participate in further study visits with a minimum time of two months between visits.

#### Data collection

Participant demographics and date of visits were collected for both studies using the REDCap (Research Electronic Data Capture) system.

#### Assays

COVID warriors and ‘STORY’ used a variety of different assays when undertaking seroepidemiological surveys, which included Roche Elecsys Anti-SARS-CoV-2 IgG spike assay, DiaSorin LIAISON SARS CoV-2 S1/S2 IgG assay, Roche Elecsys Anti-SARS-CoV-2 IgG nucleocapsid assay and UKHSA Receptor binding domain (RBD) assay.^8^^,^^9^ For each sample, one or more assay result was available but, there was no assay that was used consistently for all participants across the two studies. The overall sensitivity ranged from 64 to 96%, whereas specificity was high across all the assays (98–100%) used in the initial seroepidemiology work.

#### Sample processing

Serum samples for both COVID Warriors and STORY were stored at −80°C. The COVID Warrior samples had undergone one freeze-thaw cycle before testing on the MSD platform, while most STORY samples were frozen directly after centrifugation. No sample underwent more than two freeze-thaw cycles. Nine individual assays were run on a total of 14 plates. Each plate had an MSD standard and the percentage recovery required was close to 100% for at least 4–5 dilutions. No significant difference in assay results was identified between the plates. Over multiple studies using the MSD assay no significant batch effect was detected.

### Quantification and statistical analysis

Statistical analysis was performed by Stata Statistical Software 17 software. To compare group responses to human coronaviruses, descriptive statistics were used. This included mean, median and range, which were used to describe the age distribution of participants within each group. Groups were compared using the Mann Whitney U test as data were not normally distributed, and results with a value of p < 0.01 were considered statistically significant due to multiple comparisons (Figures 2 and 3).

To analyze the concentration of antibody over time, participants' results were plotted graphically. A mixed-effects linear regression was carried out with log-transformed antibody concentration as the outcome, fixed effects for time from the highest positive result as a continuous variable, age group and study, and random intercepts for each participant. Antibody decline was presented graphically by plotting adjusted means for antibody concentration over time with day 0 as the highest positive result (Figures 4 and 5).
