# Supplementary material for: Serum HCoV-spike specific antibodies do not protect against subsequent SARS-CoV-2 infection in children and adolescents
Source: iScience. 2023 Nov 21;26(12):108500. doi: 10.1016/j.isci.2023.108500 (PMC10711458; doi:10.1016/j.isci.2023.108500)
Supplement: Document S1. Figures S1–S5 and Table S1–S3 [file mmc1.pdf]

iScience, Volume *26*

Supplemental information

Serum HCoV-spike specific antibodies do not protect against subsequent SARS-CoV-2 infection in children and adolescents

Helen Ratcliffe, Karen S. Tiley, Stephanie Longet, Claire Tonry, Cathal Roarty, Chris Watson, Gayatri Amirthalingam, Iason Vichos, Ella Morey, Naomi L. Douglas, Spyridoula Marinou, Emma Plested, Parvinder K. Aley, Eva Galiza, Saul N. Faust, Stephen Hughes, Clare Murray, Marion R. Roderick, Fiona Shackley, Sam Oddie, Tim W.R. Lee, David P.J. Turner, Mala Raman, Stephen Owens, Paul J. Turner, Helen Cockerill, Jamie Lopez Bernal, Samreen Ijaz, John Poh, Justin Shute, Ezra Linley, Ray Borrow, Katja Hoschler, Kevin E. Brown, Miles W. Carroll, Paul Klenerman, Susanna J. Dunachie, Mary Ramsay, Merryn Voysey, Thomas Waterfield, and Matthew D. Snape

Supplementary materials

**Serum assays**	**Threshold for positivity**		**Overall sensitivity**	**Overall specificity**	**Study**
Roche Elecsys® Anti-SARS-CoV-2 IgG spike assay	≥0.8 U/ml		95.5% (95%CI 93.2-97.1)[14]	100% (95% CI 99.1-100)[14]	COVID warriors STORY
DiaSorin LIAISON® SARS CoV-2 S1/S2 IgG assay	≥ 15.0AU/ml		64.0% (95%CI 53.8-73.4) [15]	97.7% (95% CI 95.8-99.0)[15]	COVID warriors
Roche Elecsys® Anti-SARS-CoV-2 IgG nucleocapsid assay	≥1 COI		83.9% (95%CI 74.8-90.7)[16]	100% (95% CI 99.1-100)[16]	COVID warriors STORY
UKHSA Receptor binding domain (RBD) assay	≥5 T/N ratio		92.9% (95% CI 88.1-96.3) [17]	98.97% 95% CI 97.0-98.8)[17]	STORY
	Adult	Paediatric			
MSD SARS-CoV-2 S	450 AU/ml	1160 AU/ml	90.8 % (86.0–94.1)[18]	97.4 % (95%CI 94.1 to 98.9)[18]	COVID warriors STORY
MSD SARS-CoV-2 RBD	815 AU/ml	1169 AU/ml	78.1% (71.8–83.3)[18]	92.3% (95%CI 87.6 to 95.3)[18]	COVID warriors STORY
MSD SARS-CoV-2 N	3364 AU/ml	3874 AU/ml	73.0% (66.3–78.7)[18]	92.8 % (95%CI 88.2 to 95.7)[18]	COVID warriors STORY
Oral fluid assays	Threshold of positivity				
SARS-CoV-2 viral nucleoprotein (NP)	1 COI		78% (68%–86%)[13]	99% (98%–100%)[13]	STORY
SARS-CoV-2 viral spike (S)	1 COI		80% (71%–80%)[13]	99% (98%–100%)[13]	STORY

Table S1 SARS-CoV-2 antibody assays with manufacturers thresholds for positivity. Overall sensitivity and specificity data has been derived from adult data except for the oral fluid assays where the sensitivity and specificity shown was calculated from a paediatric population[13]. Related to figures 2 and 3.

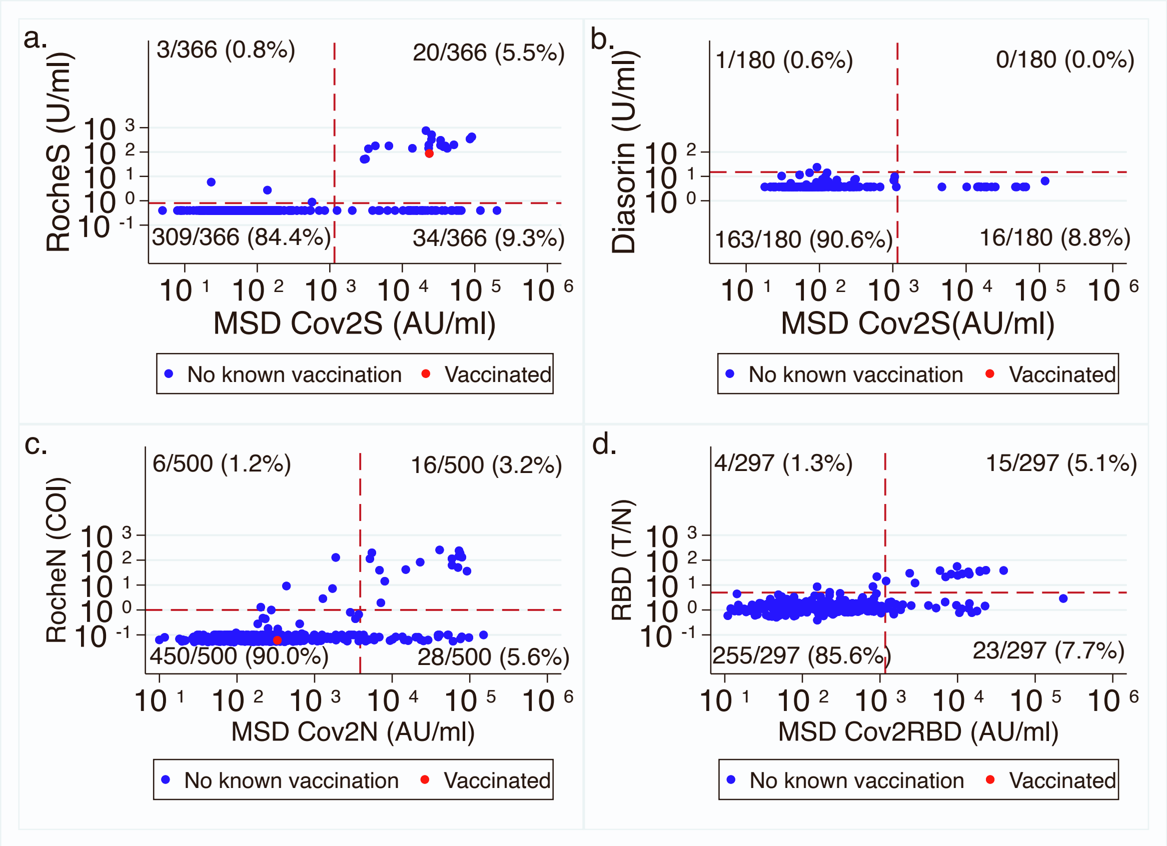

Figure S1 A comparison of assays by antigen (related to Figures 2 and 3)

a. Comparing spike assays (RocheS and MSD Cov2S) b. Comparing spike assays (Diasorin and MSD Cov2S) c. Comparing nucleocapsid assays (RocheN and MSD Cov2N). d Comparing receptor binding domain assays (in house PHE assay and MSDCov2RBD). Red markers are a sample from participants who were immunised at the time of sampling. Red dashed lines show the threshold of positivity for each assay.

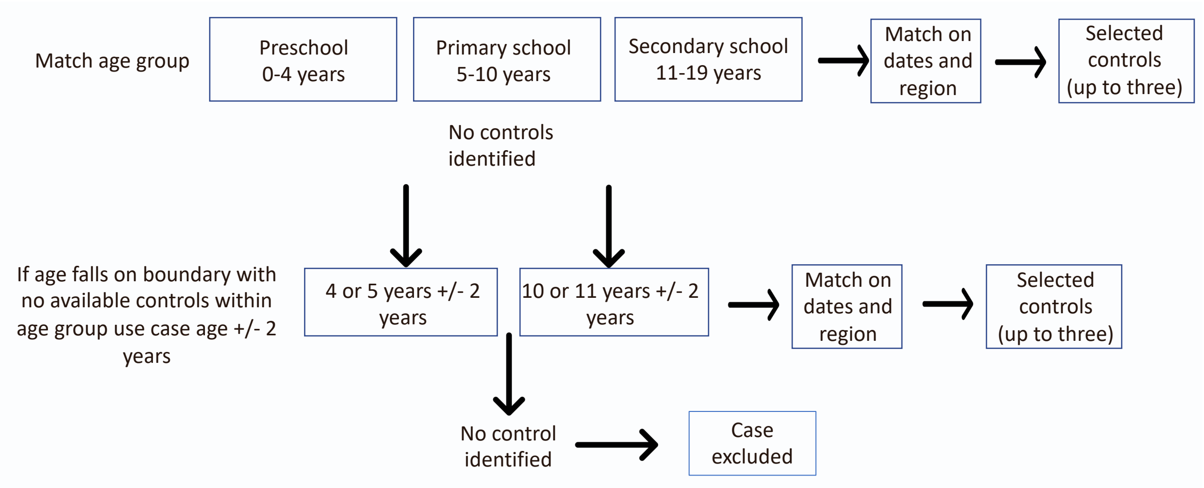

Figure S2 Age matching within the 'What's the STORY?' dataset (related to results heading *hCoV antibodies do not confer protection against SARS-CoV-2 infection)*

		Visit 1 (baseline) Odds ratio (OR) (99%CI)	p-value
SARS-CoV-2	SARS-CoV-2 Spike	2.7 (0.8-9.9)	0.05
	SARS-CoV-2 Nucleocapsid	2.0 (0.7-5.6)	0.1
	SARS-CoV-2 RBD	1.6 (0.5-4.5)	0.3
Alphacoronaviruses	229E Spike	1.0 (0.6-1.8)	0.9
	NL63 Spike	1.3 (0.4-4.0)	0.5
Betacoronaviruses	HKU1 Spike	1.9 (0.6-6.3)	0.2
	OC43 Spike	1.6 (0.5-4.7)	0.3
	SARS-CoV-1 Spike	1.8 (0.6-5.5)	0.2
	MERS Spike	1.2 (0.4-3.1)	0.7

Table S2 Shows the odds of being a case per 10-fold higher baseline titre (V1). Related to Figure 3.

	COVID warriors	COVID warriors	STORY	STORY	STORY
	2 timepoints	3 timepoints	2 timepoints	3 timepoints	4 timepoints
Age					
0-4 years	82	58	90	37	14
5-9 years	314	251	119	56	28
10-14 years	370	285	161	84	31
15-19 years	82	68	112	61	31
20-24 years			20	7	2
Sex (Male)	416	390	248	122	106
RocheN	790	661	473	219	87
RocheS	66	68	473	218	87
RBD			351	151	24
Diasorin	871	693			

Table S3 Number of participants with repeat samples by number of time points age group and assay deployed (related to Figure 4)

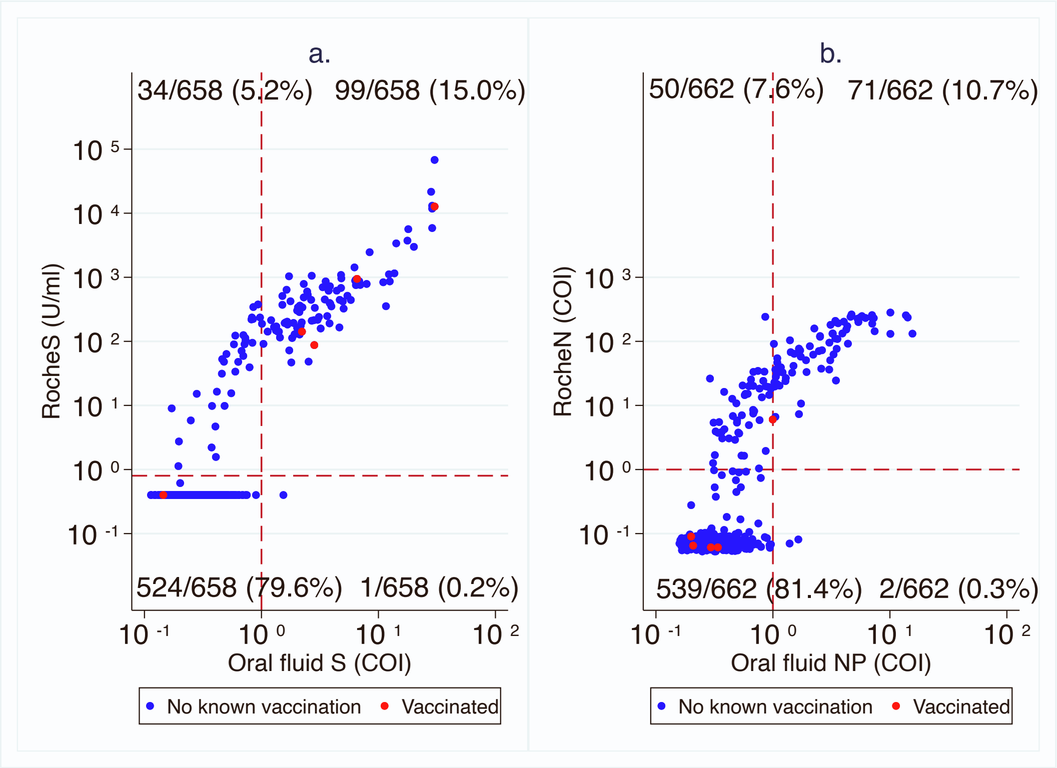

Figure S3 Matched serum and oral fluid samples for IgG against a. SARS-CoV-2 viral Spike protein and b. SARS-CoV-2 viral nucleoprotein (NP). Red markers indicate a sample from a participant who was immunised at the time of sampling. Red dashed lines show the threshold of positivity for each assay. Related to Figure 5.

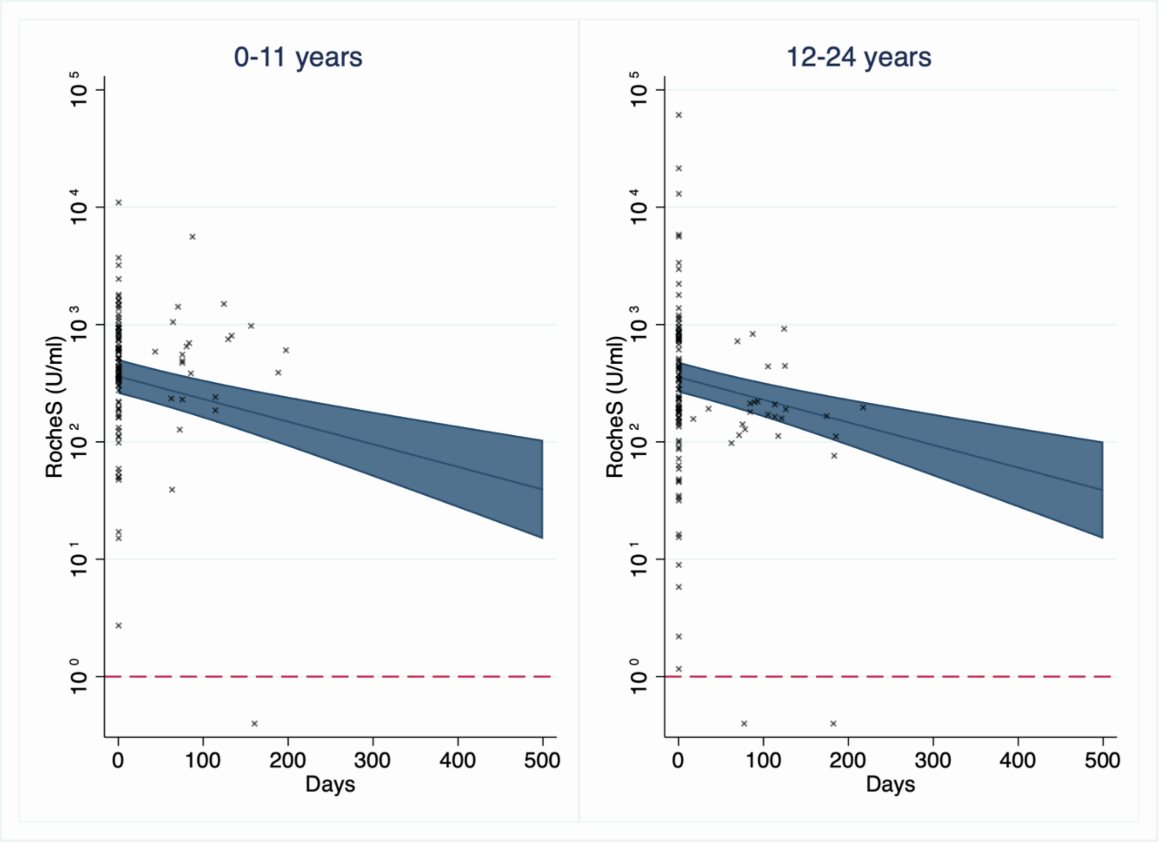

Figure S4 Mixed regression model predicting the adjusted means with time point 0 being the highest positive result with individual participant's serum antibody concentrations over time marked with (x) for Roche Elecsys® Anti-SARS-CoV-2 IgG spike antibodies for 0-11 years (a) and 12-24 years (b). 95% confidence intervals are shown. The threshold of positivity is indicated by the red line on each plot. (related to Figure 4)

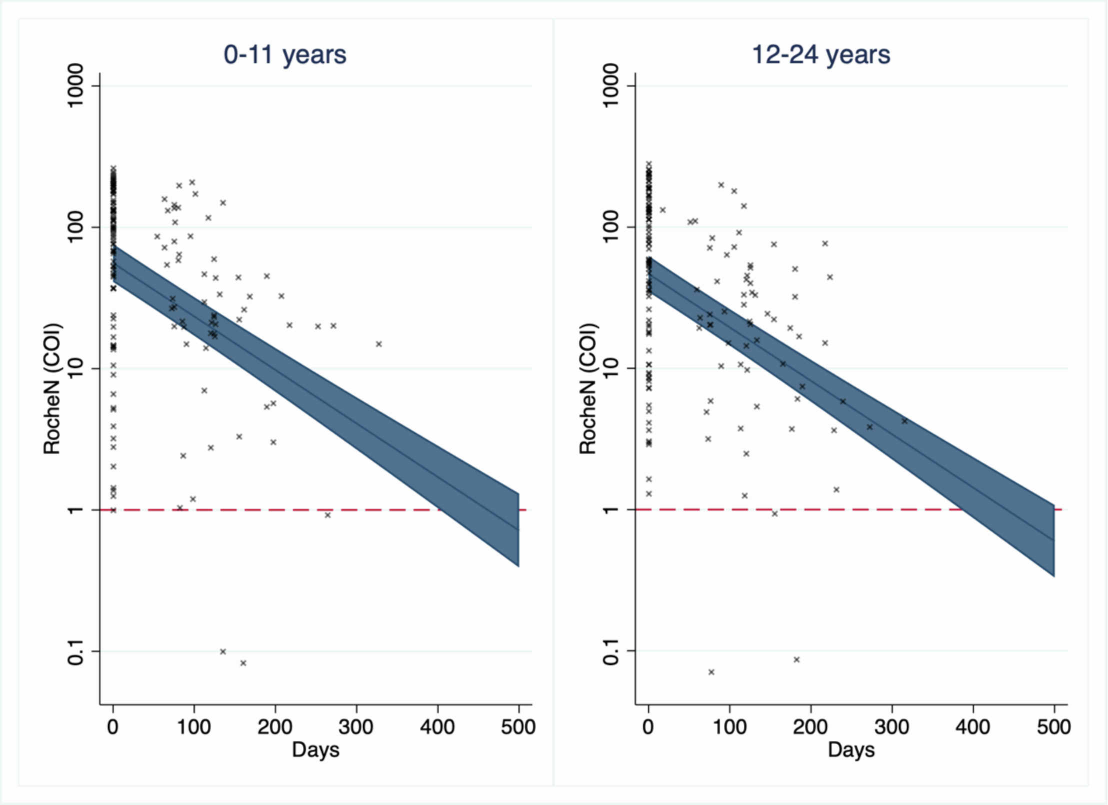

Figure S5 Mixed regression model predicting the adjusted means with time point 0 being the highest positive result with individual participant's serum antibody concentrations over time marked with (x) for Roche Elecsys® Anti-SARS-CoV-2 IgG nucleocapsid antibodies for 0-11 years (a) and 12-24 years (b). 95% confidence intervals are shown. The threshold of positivity is indicated by the red line on each plot.
